# Sex Differences in Neuropsychiatric Symptoms of Alzheimer's Disease: The Modifying Effect of Apolipoprotein E *ε*4 Status

**DOI:** 10.1155/2015/275256

**Published:** 2015-10-11

**Authors:** Yi Xing, Yi Tang, Jianping Jia

**Affiliations:** ^1^Department of Neurology, Xuan Wu Hospital, Capital Medical University, Beijing 100053, China; ^2^Center of Alzheimer's Disease, Beijing Institute for Brain Disorders, Beijing 100069, China; ^3^Beijing Key Laboratory of Geriatric Cognitive Disorders, Beijing 100053, China; ^4^Neurodegenerative Laboratory of Ministry of Education of the People's Republic of China, Beijing 100053, China

## Abstract

Sex differences in neuropsychiatric symptoms of Alzheimer's disease (AD) have been demonstrated in previous studies, and apolipoprotein E (ApoE) *ε*4 status influences psychiatric manifestations of AD. However, whether ApoE *ε*4 status modifies the sex differences in neuropsychiatric symptoms of AD is still unclear. In this study, sex differences in neuropsychiatric abnormalities were stratified and analyzed by ApoE *ε*4 status in mild AD and moderate to severe AD separately. The Clinical Dementia Rating (CDR) scale and the Neuropsychiatric Inventory (NPI) were used to assess dementia severity and neuropsychiatric symptoms. No sex differences were found in mild AD. In moderate to severe AD, among *ε*4 positive individuals, disinhibition was significantly more prevalent (8.0% in men versus 43.2% in women, *p* = 0.003) and severer (*p* = 0.003) in female patients. The frequency (16.0% in men versus 51.4% in women, *p* = 0.005) and score (*p* = 0.004) of irritability were of borderline significance after strict Bonferroni correction. In conclusion, this study supported the modifying effect of ApoE *ε*4 status on sex differences in neuropsychiatric symptoms of AD, and this modifying effect was pronounced in moderate to severe stage of AD. The interaction between gender and ApoE *ε*4 status should be considered in studies on neuropsychiatric symptoms of AD.

## 1. Introduction

Although Alzheimer's disease (AD), as the most common dementia and the major cause for senile dementia, is usually characterized by cognitive impairments, neuropsychiatric symptoms affect most of patients with AD [1]. Neuropsychiatric symptoms are significantly associated with decreased quality of patients' life [2], the heavy burden on caregivers [3], rapid cognitive decline, increased risk of institutionalization, and low survival rate of patients with AD [4, 5]. Sex difference is a common phenomenon in AD and manifests in many ways, and some previous studies had suggested sex-specific neuropsychiatric symptoms in AD. It was reported that male patients with AD were more frequently to exhibit apathy and anxiety, while delusion was more common in female patients [6–8]. The sex differences in neuropsychiatric symptoms also influence the decision of treatment, and male patients are more likely to receive antipsychotic medications [9].

Apolipoprotein E (ApoE) *ε*4 allele, as a generally acknowledged genetic risk factor for AD, extensively influences the clinical manifestations of AD, as well as neuropsychiatric symptoms. The associations between ApoE genotype and delusion, aggression, anxiety, apathy, and depression symptoms of AD have been reported [10–12]. Interestingly, the influences of ApoE *ε*4 allele on AD are more pronounced in females than in males [13]. Our previous study also suggested that ApoE *ε*4 status regulated the effects of sex hormones on neuropsychiatric symptoms of AD in female patients but not in males [14]. Thus, we inferred that ApoE *ε*4 status influences sex differences in neuropsychiatric symptoms of AD. However, this aspect still lacks systematic studies.

In this study, we investigated the interactions between gender and ApoE *ε*4 status in neuropsychiatric symptoms of AD. Sex differences in neuropsychiatric abnormalities of AD were stratified and analyzed by ApoE *ε*4 status. Considering that dementia severity influences sex differences in neuropsychiatric symptoms [9], gender comparisons were conducted in mild AD and moderate to severe AD separately.

## 2. Materials and Methods

### 2.1. Subjects

All subjects were selected from consecutive patients diagnosed with AD in the baseline stage of China Cognition and Aging Study (China COAST), which is a national study on the mild cognitive impairment (MCI) and dementia based on hospital population [14, 15]. The diagnosis of dementia was based on the Diagnostic and Statistical Manual of Mental Disorders, 4th edition (DSM-IV), criteria. Patients diagnosed with AD met the criteria of the National Institute of Neurological and Communicative Disorders and Stroke-Alzheimer's Disease and Related Disorders Association (NINCDS-ADRDA) for probable AD. Interrater reliability for cognitive tests and diagnosis was required to exceed 0.90 with videotaped interviews in China COAST. Written informed consent was obtained from all participants or their relatives. This study was approved by the Institutional Review Board of Xuan Wu Hospital.

### 2.2. Assessments

All the participants in the present study underwent the following cognitive and neuropsychiatric assessments. The Mini-Mental State Examination (MMSE) [16] and the Clinical Dementia Rating (CDR) scale [17] were used to assess global cognitive ability and dementia severity. We used the Neuropsychiatric Inventory (NPI) to determine neuropsychiatric symptoms [18]. The scoring of NPI was based on the information from the caregivers. The NPI includes the following symptoms: delusions, hallucinations, agitation/aggression, apathy, anxiety, depression, euphoria, disinhibition, irritability, aberrant motor behavior, sleep behavior disturbances, and appetite abnormalities. If a patient did not have any of these symptoms in the last month, the NPI score was 0. If the answer was “yes,” then the frequency and severity were asked. The score of each symptom was calculated as the product of the frequency and severity (maximum score = 12).

The ApoE genotypes were determined using the restriction enzyme digestion approach previously described [19]. Subjects were classified as ApoE *ε*4 positive if they carried at least one copy of the *ε*4 allele.

### 2.3. Statistical Analysis

To summarize demographic data of our patients, we used *χ* ^2^ tests or Fisher's exact tests if needed for dichotomous variables and independent sample *t*-tests for continuous data. We analyzed gender differences among patients with different dementia severities separately with similar analytic strategies. Patients were classified into mild dementia (CDR = 1) and moderate to severe dementia (CDR = 2 or 3) according to the CDR scores. Mann-Whitney *U* tests were used to compare sex differences in the total NPI score and each NPI item score. Sex differences in the prevalence of each NPI subscale (present, a score of 1 or higher; not present, a score of 0) were examined using *χ* ^2^ tests or Fisher's exact tests. Logistic regression analyses were performed to control for age and educational duration. The individual symptoms were dependent variable, and sex, ApoE *ε*4 status, and the interaction term (ApoE *ε*4 status × sex) were added to regression models as independent variables. A *p* value < 0.05 was regarded as statistically significant. For multiple comparisons, the *α* level was set at 0.004 (0.05/12) in accordance with the Bonferroni adjustment.

## 3. Results

### 3.1. Patients' Characteristics

A total of 315 patients were included in our study, including 158 mild AD patients (CDR = 1) and 157 moderate to severe AD patients (113 with the CDR = 2 and 46 with the CDR = 3). The characteristics of our subjects are presented in Table 1. Male patients had a significantly higher educational level than female patients. None of all subjects had the ApoE genotype of *ε*2/*ε*2. In mild AD, male patients had significantly higher frequencies of *ε*3/*ε*3 genotype than female patients, while females had higher *ε*3/*ε*4 frequencies than males. In moderate to severe AD, there was no sex difference in ApoE genotype frequencies.

### 3.2. Sex Differences in the Scores and Frequencies of Neuropsychiatric Symptoms

Table 2 shows the gender comparisons of the prevalence and scores of individual NPI symptoms. In mild AD, 74.3% of men and 70.2% of women reported at least one neuropsychiatric symptom. There were no sex differences in either scores or frequencies of neuropsychiatric symptoms in mild AD, even after stratified analysis by ApoE *ε*4 status. In moderate to severe AD, 81.5% male patients and 90.2% female patients had neuropsychiatric symptoms. In all the moderate to severe AD patients, sex differences were not found. However, in *ε*4 positive group, disinhibition was significantly more prevalent in female patients (8.0% in men versus 43.2% in women, *p* = 0.003), and the score of disinhibition (*p* = 0.003) was also significantly higher in females. The prevalence (16.0% in men versus 51.4% in women, *p* = 0.005) and score (*p* = 0.004) of irritability were of borderline significance after strict Bonferroni correction.

After controlling for age and educational duration, the logistic regression analyses demonstrated that the ApoE *ε*4 status × sex interaction was associated with disinhibition and irritability in moderate to severe AD. Compared to other patients in moderate to severe stage, those female patients carrying *ε*4 allele were 7.7 times (95% CI 1.09–54.5, *p* = 0.040) and 8.3 times (95% CI 1.64–42.1, *p* = 0.010) more likely to have disinhibition and irritability, respectively.

## 4. Discussion

In this study, we systematically investigated the sex differences in neuropsychiatric symptoms in mild AD and moderate to severe AD, and we analyzed the modifying effect of ApoE *ε*4 status. Our results demonstrated that, before stratified analysis by ApoE *ε*4 status, there were no sex differences in neuropsychiatric symptoms. However, in *ε*4 positive individuals, female patients had significantly higher frequency and score of disinhibition than male patients in moderate to severe AD even after strict Bonferroni correction. For irritability, after Bonferroni correction, our study only confirmed a borderline significance, which needs to be further investigated. It was suggested that female patients with at least one copy of the *ε*4 allele were significantly more likely to have some neuropsychiatric symptoms in moderate to severe AD.

Consistent with previous studies [6, 7], gender differences were not significant in the overall prevalence and severity of NPI symptoms in our study. With respect to individual symptoms, some previous studies suggested there were sex differences in apathy [6], delusions [7], and anxiety [8] in AD. However, before stratified analysis according to ApoE *ε*4 status, no sex differences were found in this study. The discrepancies of these results were probably attributed to different study subjects and approaches. The demographics of subjects, including ethnicity and age, may influence the onset of neuropsychiatric symptoms [7]. The differences in dementia severities of participants may also cause the inconsistencies between studies [8]. Furthermore, the different instruments used to evaluate neuropsychiatric symptoms, other than NPI [6], may also partially explain the diverse results.

The underlying pathophysiological mechanisms of neuropsychiatric symptoms of AD are still not completely clear. However, increasing evidence has suggested that some pathological or neuroimaging biomarkers of AD were associated with neuropsychiatric disorders. Interestingly, it was also suggested that there was an interaction between sex and ApoE *ε*4 status on these biomarkers of AD. In pathological and CSF biomarkers, tau phosphorylation had been reported to be accelerated in AD with psychosis [20], and the increase of CSF concentration of amyloid *β* protein (A*β*) was related to the presence of agitation and irritability [21]. Correspondingly, females with ApoE *ε*4 allele were found to have greater A*β* and neurofibrillary tangle in autopsy cases [22] and higher CSF levels of tau in healthy elderly adults [23]. In terms of neuroimaging biomarkers, it was reported that the atrophy of hippocampal region was associated with agitation and aggression in AD [24], and the amygdala atrophy, which was comparable to hippocampal atrophy, was potentially related to irritability [25]. Meantime, previous studies have showed that the presence of ApoE *ε*4 allele was associated with smaller hippocampal volumes in women than in men in mild cognitive impairment (MCI) and AD [26]. Psychological symptoms in AD were also associated with white matter hyperintensities (WMH), and the disinhibition symptom was related to lower WMH volume [27]. Interestingly, female ApoE *ε*4 carriers had significantly reduction of white matter integrity of the tract connecting the hippocampus [28]. In addition, compared to females without ApoE *ε*4 and male carriers, females with ApoE *ε*4 allele had significantly reduced default mode connectivity [23], which was associated with neuropsychiatric disorders and reduced in AD patients [29, 30]. All these lines of evidence suggested that ApoE *ε*4 allele may have important modifying effects on gender-specific manifestations of neuropsychiatric symptoms.

There are some limitations in our study. Our subjects were chosen from neurology outpatients and therefore were not representative of the general population, though our study results might be of value in clinical setting. Furthermore, although the NPI we used is a validated and widely used instrument, it relies on the information from caregivers instead of patients.

## 5. Conclusions

This study supported the modifying effect of ApoE *ε*4 status on sex differences in neuropsychiatric symptoms of AD, and this modifying effect was pronounced in moderate to severe stage of AD. The interaction between sex and ApoE *ε*4 status should be considered in further studies on neuropsychiatric symptoms of AD.

## Figures and Tables

**Table 1:** Characteristics of subjects and ApoE genotype frequencies.

	Mild AD			Moderate to severe AD		
	Male	Female	*p* value	Male	Female	*p* value
	(*n* = 74)	(*n* = 84)		(*n* = 65)	(*n* = 92)	
Age	71.0 (9.1)	70.9 (10.0)	0.967	69.0 (10.1)	66.7 (10.6)	0.156
Education (yr)	9.2 (4.6)	6.4 (5.4)	**0.001**	8.0 (4.6)	4.8 (4.4)	**<0.001**
MMSE	17.9 (5.6)	17.1 (4.9)	0.294	12.8 (6.0)	12.0 (5.1)	0.803
ApoE genotype^*∗*^						
*ε*4 negative	56 (75.7)	46 (54.8)	**0.006**	40 (61.5)	55 (59.8)	0.825
*ε*2/*ε*3	7 (9.5)	9 (10.7)	0.794	6 (9.2)	5 (9.1)	0.365
*ε*3/*ε*3	49 (66.2)	37 (44.0)	**0.005**	34 (52.3)	50 (54.3)	0.801
*ε*4 positive	18 (24.3)	38 (45.2)	**0.006**	25 (38.5)	37 (40.2)	0.825
*ε*2/*ε*4	0	1 (1)	1.000	1 (1.5)	2 (2.2)	1.000
*ε*3/*ε*4	14 (18.9)	34 (40.5)	**0.003**	19 (29.2)	30 (32.6)	0.653
*ε*4/*ε*4	4 (5.4)	3 (3.6)	0.707	5 (7.7)	5 (5.4)	0.742

^*∗*^Values are presented as numbers (percentages).

**Table 2:** The gender comparisons of the frequencies and scores of neuropsychiatric symptoms.

NPI items	Mild AD						Moderate to severe AD					
	All		*ε*4 negative		*ε*4 positive		All		*ε*4 negative		*ε*4 positive	
	M	F	M	F	M	F	M	F	M	F	M	F
	*n* = 74	*n* = 84	*n* = 56	*n* = 46	*n* = 18	*n* = 38	*n* = 65	*n* = 92	*n* = 40	*n* = 55	*n* = 25	*n* = 37
Delusions	16.2	26.2	12.5	21.7	27.8	31.6	33.8	39.1	37.5	41.8	28.0	35.1
	0.7 ± 2.3	1.3 ± 4.1	0.5 ± 1.9	0.8 ± 1.9	1.3 ± 3.2	1.9 ± 5.8	2.1 ± 3.9	2.0 ± 3.7	2.3 ± 4.0	1.9 ± 3.4	1.8 ± 3.9	2.3 ± 4.3

Hallucinations	10.8	15.5	8.9	8.7	16.7	23.7	24.6	26.1	25.0	21.8	24.0	32.4
	0.3 ± 1.3	0.8 ± 2.6	0.2 ± 1.1	0.4 ± 1.5	0.6 ± 1.9	1.3 ± 3.4	1.3 ± 3.3	1.5 ± 3.3	1.4 ± 3.2	1.2 ± 3.0	1.2 ± 3.3	2.0 ± 3.9

Agitation/ aggression	20.3	20.2	17.8	17.4	27.8	23.7	32.3	35.9	37.5	30.9	24.0	43.2
	0.7 ± 2.0	1.1 ± 2.8	0.7 ± 2.1	0.9 ± 2.5	0.8 ± 1.6	1.2 ± 3.2	1.5 ± 3.2	1.8 ± 3.4	1.5 ± 3.1	1.3 ± 3.1	1.4 ± 3.5	2.5 ± 3.9

Depression	28.4	32.1	26.8	34.8	33.3	28.9	40.0	46.7	42.5	45.4	36.0	48.6
	1.1 ± 2.4	1.7 ± 3.7	1.1 ± 2.6	1.5 ± 2.8	1.0 ± 1.7	1.9 ± 4.6	1.3 ± 2.6	1.9 ± 3.0	1.4 ± 2.9	1.9 ± 2.8	1.3 ± 3.0	2.0 ± 3.2

Anxiety	18.9	19.0	19.6	19.6	16.7	18.4	35.4	45.7	32.5	43.6	40.0	48.6
	0.7 ± 2.1	0.9 ± 2.6	0.8 ± 2.4	1.1 ± 2.8	0.4 ± 1.0	0.8 ± 2.4	1.8 ± 3.6	2.5 ± 3.7	0.7 ± 2.2	1.8 ± 3.0	2.4 ± 4.4	3.5 ± 4.7

Euphoria	6.7	3.6	7.1	4.3	5.6	2.6	10.7	5.4	17.5	7.3	0	2.7
	0.3 ± 1.2	0.1 ± 0.7	0.3 ± 1.3	0.2 ± 0.8	0.2 ± 0.9	0.1 ± 0.6	0.4 ± 1.7	0.3 ± 1.5	2.8 ± 4.0	0.4 ± 1.5	—	0.2 ± 1.3

Apathy	36.5	27.4	35.7	32.6	38.9	21.1	52.3	52.2	50.0	52.7	56.0	51.4
	1.7 ± 3.1	1.3 ± 2.9	1.7 ± 3.1	1.8 ± 3.5	1.8 ± 3.3	0.7 ± 1.5	3.3 ± 4.4	3.0 ± 4.2	1.0 ± 2.7	2.8 ± 4.1	4.0 ± 5.0	3.3 ± 4.3

Disinhibition	5.4	10.7	5.3	13.0	5.6	7.9	12.3	27.2	15.0	16.4	**8.0** ^**∗∗**^	**43.2** ^**∗∗**^
	0.1 ± 0.5	0.5 ± 2.0	0.1 ± 0.6	0.7 ± 2.6	0.1 ± 0.2	0.2 ± 0.9	0.7 ± 2.2	1.2 ± 2.6	1.0 ± 2.7	0.6 ± 1.9	**0.3 ± 1.2** ^††^	**2.1 ± 3.3** ^††^

Irritability	14.7	16.7	16.1	8.7	11.1	26.3	20.0	30.4	22.5	16.4	**16.0** ^*∗*^	**51.4** ^**∗**^
	0.9 ± 2.6	0.6 ± 1.8	0.9 ± 2.7	0.2 ± 1.0	0.9 ± 2.6	0.9 ± 2.4	1.3 ± 3.3	1.7 ± 3.4	1.6 ± 3.7	0.9 ± 2.8	**0.8 ± 2.5** ^†^	**3.0 ± 3.9** ^†^

Aberrant motor behavior	13.5	16.7	12.5	21.7	16.7	10.5	24.6	38.0	35.0	34.5	28.0	43.2
	0.6 ± 2.0	1.0 ± 2.8	0.6 ± 2.1	1.4 ± 3.2	0.6 ± 1.6	0.4 ± 2.0	1.7 ± 3.7	2.3 ± 4.0	1.6 ± 3.7	2.0 ± 4.1	2.0 ± 3.8	2.7 ± 4.3

Sleep behavior disturbances	16.2	13.1	14.3	10.9	22.2	15.8	30.8	28.3	35.0	23.6	24.0	35.1
	0.7 ± 1.9	0.9 ± 2.7	0.6 ± 1.7	0.7 ± 2.6	1.1 ± 2.6	1.1 ± 2.9	2.1 ± 4.0	1.6 ± 3.5	2.8 ± 4.8	1.2 ± 3.0	0.9 ± 2.1	2.3 ± 4.2

Appetite abnormalities	8.1	11.9	8.9	13.0	5.6	10.5	16.9	15.2	17.5	12.7	16.0	18.9
	0.3 ± 1.3	0.5 ± 1.8	0.3 ± 1.3	0.6 ± 2.0	0.3 ± 1.4	0.4 ± 1.5	1.3 ± 3.4	0.8 ± 2.4	1.4 ± 3.5	0.5 ± 2.0	1.2 ± 3.4	1.2 ± 2.9

Total	74.3	70.2	69.6	71.7	88.9	68.4	81.5	90.2	82.5	90.9	80.0	89.2
	8.1 ± 10.3	10.6 ± 16.7	7.8 ± 9.7	10.3 ± 13.0	9.1 ± 12.4	11.0 ± 20.3	18.6 ± 24.6	20.7 ± 21.4	19.7 ± 27.0	17.2 ± 18.9	16.8 ± 20.5	25.9 ± 24.0

Data are expressed as percentages of patients with individual symptoms and means of scores ± SDs.

Please note that the means with standard deviations of scores are represented the same as previous literatures, though the data are not normally distributed.

^*∗*^Gender differences in the prevalence of NPI symptoms, *χ* ^2^ tests, *p* < 0.05, and ^*∗∗*^ *p* < 0.004.

^†^Gender differences in NPI scores, Mann-Whitney *U* test, *p* < 0.05, and ^††^ *p* < 0.004.
